# Growth inhibition and differentiation induction in human monoblastic leukaemia cells by 1alpha-hydroxyvitamin D derivatives and their enhancement by combination with hydroxyurea.

**DOI:** 10.1038/bjc.1998.6

**Published:** 1998

**Authors:** M. Makishima, J. Okabe-Kado, Y. Honma

**Affiliations:** Department of Chemotherapy, Saitama Cancer Center Research Institute, Kita-adachi, Japan.

## Abstract

The active form of vitamin D, 1alpha,25-dihydroxyvitamin D3 (1,25(OH)2D3), is a potent inducer of differentiation in myeloid leukaemia cells, but its clinical use is limited because of its hypercalcaemic activity. We examined the ability of 1,25(OH)2D3 in combination with several anti-cancer drugs to inhibit the proliferation of, and induce differentiation in, human monoblastic leukaemia U937 cells. Hydroxyurea (HU), cytarabine and camptothecin showed effective synergism with 1,25(OH)2D3 with regard to growth inhibition, while daunorubicin and etoposide had only modest synergistic effects. HU and cytarabine effectively enhanced nitroblue tetrazolium-reducing activity induced by 1,25(OH)2D3. HU also enhanced the morphological maturation and expression of CD11b and CD14 in cells treated with 1,25(OH)2D3. Among the anti-cancer drugs examined, HU had the greatest synergistic effects with 1,25(OH)2D3 with regard to growth inhibition and differentiation induction in U937 cells. HU also enhanced the differentiation of other myeloid leukaemia HL-60, ML-1, THP-1, P39/TSU, P31/FUJ and NB4 cells induced by 1,25(OH)2D3 and that of U937 cells induced by 24-epi-1,25(OH)2D2 and 1,25(OH)2D7. Interestingly, 1alpha(OH)D derivatives (1alpha-hydroxyvitamin D3, D2, D4 and D7) effectively induced the differentiation of monoblastic leukaemia U937, P39/TSU and P31/FUJ cells. HU also enhanced the growth inhibition and differentiation of U937 cells induced by 1alpha(OH)D derivatives. As 1alpha(OH)D derivatives preferentially act on monocytic cells, they may be useful in the treatment of acute monocytic leukaemia, both alone and in combination with HU.


British Journal of Cancer (1998) 77(1), 33–39
© 1998 Cancer Research Campaign

### Growth inhibition and differentiation induction in human monoblastic leukaemia cells by 1α-hydroxyvitamin D derivatives and their enhancement by combination with hydroxyurea

**M Makishima, J Okabe-Kado and Y Honma**

Department of Chemotherapy, Saitama Cancer Center Research Institute, 818 Komuro, Ina-machi, Kita-adachi, Saitama 362, Japan

**Summary** The active form of vitamin D, 1α,25-dihydroxyvitamin $D_3$ ($1,25(OH)_2D_3$), is a potent inducer of differentiation in myeloid leukaemia cells, but its clinical use is limited because of its hypercalcaemic activity. We examined the ability of $1,25(OH)_2D_3$ in combination with several anti-cancer drugs to inhibit the proliferation of, and induce differentiation in, human monoblastic leukaemia U937 cells. Hydroxyurea (HU), cytarabine and camptothecin showed effective synergism with $1,25(OH)_2D_3$ with regard to growth inhibition, while daunorubicin and etoposide had only modest synergistic effects. HU and cytarabine effectively enhanced nitroblue tetrazolium-reducing activity induced by $1,25(OH)_2D_3$. HU also enhanced the morphological maturation and expression of CD11b and CD14 in cells treated with $1,25(OH)_2D_3$. Among the anti-cancer drugs examined, HU had the greatest synergistic effects with $1,25(OH)_2D_3$ with regard to growth inhibition and differentiation induction in U937 cells. HU also enhanced the differentiation of other myeloid leukaemia HL-60, ML-1, THP-1, P39/TSU, P31/FUJ and NB4 cells induced by $1,25(OH)_2D_3$ and that of U937 cells induced by 24-*epi*-$1,25(OH)_2D_2$ and $1,25(OH)_2D_7$. Interestingly, 1α(OH)D derivatives (1α-hydroxyvitamin $D_3$, $D_2$, $D_4$ and $D_7$) effectively induced the differentiation of monoblastic leukaemia U937, P39/TSU and P31/FUJ cells. HU also enhanced the growth inhibition and differentiation of U937 cells induced by 1α(OH)D derivatives. As 1α(OH)D derivatives preferentially act on monocytic cells, they may be useful in the treatment of acute monocytic leukaemia, both alone and in combination with HU.

**Keywords:** leukaemia; vitamin $D_2$; vitamin $D_3$; vitamin $D_4$; vitamin $D_7$; hydroxyurea

The prognosis of acute myeloid leukaemia has recently improved through the application of intensive chemotherapy and bone marrow transplantation. However, intensive chemotherapy is not used in elderly patients or in patients with hypoplastic leukaemia or myelodysplastic syndrome because of severe complications. The incidence of induction death among elderly patients in their initial induction therapy, even when supported with cytokines, is higher than 10% (Schiffer, 1996). Indications for bone marrow transplantation are limited to young patients with HLA-matched donors (Goldman, 1994). Differentiation therapy is one possible approach for surviving patients who cannot be treated with intensive chemotherapy or bone marrow transplantation.

Differentiation therapy has been used successfully to treat acute promyelocytic leukaemia (Degos et al, 1995). *All-trans* retinoic acid induces complete remission in more than 90% of patients with acute promyelocytic leukaemia with a t (15;17) chromosomal translocation. However, the use of *all-trans* retinoic acid is limited to acute promyelocytic leukaemia. Vitamin D is another potential inducer for differentiation therapy. The active form of vitamin D, 1α,25-dihydroxyvitamin $D_3$ ($1,25(OH)_2D_3$), induces differentiation in mouse and in human leukaemia cells (Abe et al, 1981; Miyaura et al, 1981) and prolongs the survival of mice inoculated with myeloid leukaemia cells (Honma et al, 1983). However, clinical trials of $1,25(OH)_2D_3$ in patients with myelodysplastic syndrome have not been successful because of hypercalcaemia (Koeffler et al, 1985). Several analogues of $1,25(OH)_2D_3$ that show anti-cancer activity and only weak activity for inducing hypercalcaemia have been developed, but they are not yet available for the clinical treatment of cancer or leukaemia (Abe et al, 1991; Pakkala et al, 1995). To overcome the adverse effects of vitamin D, we are investigating the effects of the combination of $1,25(OH)_2D_3$ with other drugs (Makishima and Honma, 1996; Makishima et al, 1996). As the combination of *all-trans* retinoic acid with low doses of anti-cancer drugs produced better results than either drug alone for the treatment of acute myeloid leukaemia (Venditti et al, 1995), in this study, we investigated the effects of the combination of $1,25(OH)_2D_3$ and its analogues with various anti-cancer drugs on growth inhibition and differentiation induction in myelomonocytic leukaemia cells.

*Received*
*Revised 1 June 1997*
*Accepted 10 June 1997*

*Correspondence to:* Y Honma

### MATERIALS AND METHODS

### Materials

1α,25-Dihydroxyvitamin $D_7$ ($1,25(OH)_2D_7$), 24-*epi*-1α,25-dihydroxyvitamin $D_2$ (24-*epi*-$1,25(OH)_2D_2$), 1α-hydroxyvitamin $D_3$ ($1\alpha(OH)D_3$), $1\alpha(OH)D_2$, $1\alpha(OH)D_4$ and $1\alpha(OH)D_7$ were synthesized (Figure 1) (Tachibana and Tsuji, 1992) and donated by the Fine Chemical Research Center, Nisshin Flour Milling (Saitama, Japan). Chlorambucil, daunorubicin, actinomycin D, hydroxyurea

**Figure 1** Chemical structures of vitamin D derivatives

(HU), cytarabine (Ara-C) and camptothecin were purchased from Sigma (St Louis, MO, USA), and $1,25(OH)_2D_3$ was from Wako Pure Chemical Industry (Osaka, Japan). Etoposide was obtained from Nippon Kayaku (Tokyo, Japan).

### Cell lines and cell culture

Human myeloid leukaemia U937, HL-60, ML-1, THP-1, P39/TSU, P31/FUJ and NB4 cells (Lanotte et al, 1991) were cultured in suspension in RPMI 1640 medium containing 10% fetal bovine serum and 80 μg $ml^{-1}$ gentamicin at 37°C in a humidified atmosphere of 5% carbon dioxide in air (Makishima et al, 1996).

### Cell growth and differentiation

Suspensions of cells were cultured with or without the test compounds in multidishes. The cells were counted in a Model ZM Coulter Counter (Coulter Electronics, Luton, UK). Nitroblue tetrazolium (NBT) reduction was assayed colorimetrically (Makishima et al, 1996). Lysozyme activity in the conditioned medium was determined using a lysoplate (Makishima et al, 1996). One unit is equivalent to 1 μg $ml^{-1}$ egg-white lysozyme. Cell morphology was examined in cell smears stained with May-Grünwald and Giemsa solutions (Merck, Darmstadt, Germany).

### Analysis of the effects of combinations of drugs

The interaction of the two compounds was quantified by determining the combination index (CI) according to the classic isobologram equation:

$$CI = D_1/Dx_1 + D_2/Dx_2$$

where Dx is the concentration of one drug alone required to produce an effect and $D_1$ and $D_2$ are the doses of compounds 1 and 2, respectively, in combination that produce the same effect (Berenbaum, 1989). Using this analysis, the combined effects of the two drugs can be assessed as being either additive (CI = 1), synergistic (CI < 1) or antagonistic (CI > 1). An isobologram was also used to determine the effect of combinations of drugs (Berenbaum, 1989). Concentration-dependent effects were determined from isoeffective concentrations for each compound and for one compound with fixed concentrations of another. The additive lines were indicated as calculated by mode I and mode II systems (Steel and Peckham, 1979).

### Flow cytometry

Expression of the granulocyte- and monocyte-specific antigens CD11b and CD14 on the cell surface was determined using indirect immunofluorescent staining and flow cytometry (Makishima and Honma, 1996). Mouse monoclonal antibodies to CD11b (2LPM19c), CD14 (TÜK4), control mouse IgG1, IgG2a and FITC-conjugated $F(ab')_2$ fragment of goat antimouse IgG were obtained from Dako (Glostrup, Denmark). The stained cells were assayed using a flow cytometer (Epics XL; Coulter Electronics)

**Table 1** Effects of the combination of anticancer drugs and $1,25(OH)_2D_3$ on growth inhibition and NBT-reducing activity of human monoblastic leukaemia U937 cells

| Drugs | $IC_{50}$ for growth suppression | | CI[b] | NBT reduction[c] ($A_{560}$ per $10^7$ cells) | | Ratio[d] |
|---|---|---|---|---|---|---|
| | $-VD_3$ | $+VD_3$[a] | | $-VD_3$ | $+VD_3$ | |
| Chlorambucil | 5.19 μM | 3.90 μM | 0.90 | 1.15 ± 0.13 | 2.14 ± 0.27 | 1.9 |
| Daunorubicin | 2.73 nM | 1.53 nM | 0.71 | 0.93 ± 0.07 | 2.17 ± 0.14 | 2.3 |
| Actinomycin D | 88.1 pM | 76.7 pM | 1.02 | 0.65 ± 0.07 | 2.06 ± 0.06 | 3.2 |
| HU | 50.7 μM | 15.8 μM | 0.46 | 1.26 ± 0.25 | 7.78 ± 0.34[e] | 6.2 |
| Ara-C | 6.86 nM | 2.41 nM | 0.50 | 1.02 ± 0.14 | 3.56 ± 0.46 | 3.5 |
| Camptothecin | 9.84 nM | 4.14 nM | 0.57 | 1.84 ± 0.16 | 3.70 ± 0.38 | 2.0 |
| Etoposide | 38.3 nM | 25.3 nM | 0.81 | 1.20 ± 0.03 | 2.82 ± 0.10 | 2.4 |
| None | | | | 0.54 ± 0.04 | 1.42 ± 0.09 | 2.6 |

Cells ($5 \times 10^4$ cells $ml^{-1}$) were treated with anti-cancer drugs in the absence or presence of $1,25(OH)_2D_3$ ($VD_3$) for 4 days. $IC_{50}$ values were determined from the means of triplicate data and values of NBT reduction represent the means ± SD of three separate experiments. [a]$IC_{50}$ values for anti-cancer drugs in the presence of $3 \times 10^{-9}$ M $1,25(OH)_2D_3$. [b]Combination index (CI) at $IC_{50}$ for growth inhibition. CI values at a fixed concentration of $1,25(OH)_2D_3$ ($3 \times 10^{-9}$ M) were calculated as described in the Materials and methods. $IC_{50}$ of $1,25(OH)_2D_3$ was $2.01 \times 10^{-8}$ M. In this assay, CI = 1 indicates an additive effect, CI <1 indicates synergism and CI >1 indicates antagonism. [c]NBT reduction in the cells treated with anti-cancer drugs at their $IC_{50}$ for growth inhibition in the absence or presence of $3 \times 10^{-9}$ M $1,25(OH)_2D_3$. $1,25(OH)_2D_3$ at the $IC_{50}$ induced the activity to 4.64 $A_{560}$. [d]Ratio of the NBT reduction: $+1,25(OH)_2D_3$ / $-1,25(OH)_2D_3$. [e]$P$<0.0005 compared with other anti-cancer drugs plus $1,25(OH)_2D_3$.

© Cancer Research Campaign 1998

**Figure 2** Effects of the combination of HU and $1,25(OH)_2D_3$ with regard to growth inhibition and differentiation induction in human monoblastic leukaemia U937 cells. (**A**) Isobologram for $1,25(OH)_2D_3$ and HU at the $IC_{50}$ for growth inhibition. The dashed lines I and II indicate additive interaction calculated by mode I and II systems respectively (Steel and Peckham, 1979). In mode II, when the dose of HU is chosen, an isoeffect curve is calculated by taking the dose increment of $1,25(OH)_2D_3$ that gives the required contribution to $IC_{50}$, estimating how much HU reduces the requirement of $1,25(OH)_2D_3$. (**B**) NBT-reducing activities of cells treated with a combination of HU and 0 (○), $3 \times 10^{-10}$ M (●), $3 \times 10^{-9}$ M (▲) or $3 \times 10^{-8}$ M (■) $1,25(OH)_2D_3$. (**C**) CD11b expression by cells treated with a combination of HU and 0 (●), $3 \times 10^{-10}$ M (▲) or $3 \times 10^{-9}$ M (■) $1,25(OH)_2D_3$. $1,25(OH)_2D_3$ at $3 \times 10^{-6}$ M induced the expression to 6.41 units. Cells ($5 \times 10^4$ cells $ml^{-1}$) were cultured with test compounds for 4 days. Values represent the means (± SD) of three separate experiments and the mean fluorescence intensity of fluorescence-positive cells was calculated using the Immuno-4 histogram analysis program (Coulter), with mouse immunoglobulin of the same isotype as a negative control. The Immuno-4 program subtracts a control histogram from a test histogram to calculate the mean fluorescence intensity in the test histogram (Overton, 1988).

### Statistical evaluation

Statistical analyses were performed using an unpaired two-tailed Student's *t*-test.

### RESULTS

### Effects of the combination of anti-cancer drugs with $1,25(OH)_2D_3$ on the growth and differentiation of human monoblastic leukaemia U937 cells

We examined several anti-cancer drugs in combination with $1,25(OH)_2D_3$ to determine the effects on growth inhibition in human monoblastic leukaemia U937 cells. Chlorambucil is an alkylating agent; daunorubicin and actinomycin D are antibiotics; HU and Ara-C are inhibitors of nucleotide metabolism; and camptothecin and etoposide are inhibitors of topoisomerases. These drugs all inhibited the proliferation of U937 cells concentration dependently; their $IC_{50}$ values are indicated in Table 1. The effects of the combination of anti-cancer drugs and $1,25(OH)_2D_3$ were determined using the CI calculated from the $IC_{50}$ values of anti-cancer drugs in the presence of $3 \times 10^{-9}$ M $1,25(OH)_2D_3$. HU inhibited the proliferation of U937 cells at an $IC_{50}$ of 50.7 μM in the absence of $1,25(OH)_2D_3$ and at an $IC_{50}$ of 15.8 μM in its presence (CI = 0.46, indicating synergism). The confidence intervals (CIs) for Ara-C, camptothecin, daunorubicin and etoposide were 0.50, 0.57, 0.71 and 0.81, respectively, also indicating synergism. The combinations of chlorambucil and actinomycin D with $1,25(OH)_2D_3$ were additive.

We examined the effects of anti-cancer drugs in combination with $1,25(OH)_2D_3$ on NBT-reducing activity, a typical marker of myelomonocytic differentiation, in U937 cells. The anti-cancer drugs showed only weak activity for inducing NBT reduction (Table 1). Next, the NBT-reducing activity induced by anti-cancer drugs in combination with $3 \times 10^{-9}$ M $1,25(OH)_2D_3$ was examined. HU plus $1,25(OH)_2D_3$ effectively increased the activity 6.2-fold from HU alone and 5.5-fold from $1,25(OH)_2D_3$ alone (Table 1). Camptothecin and Ara-C in combination with $1,25(OH)_2D_3$ modestly induced this activity. Among the anti-cancer drugs we examined, HU had the greatest synergistic effect with $1,25(OH)_2D_3$ for growth inhibition and induction of NBT-reducing activity in U937 cells.

### Effects of HU plus $1,25(OH)_2D_3$ on growth inhibition and differentiation induction in human myelomonocytic leukaemia cells

The concentration-dependent effects of the combination of HU with $1,25(OH)_2D_3$ on U937 cells were examined. Isoboles for growth inhibition show that their combination is synergistic and the presence of HU markedly reduced effective concentrations of $1,25(OH)_2D_3$ (Figure 2A). HU up to 75 μM induced NBT-reducing activity of U937 cells only slightly (Figure 2B). While $1,25(OH)_2D_3$ at $3 \times 10^{-10}$ M did not induce NBT-reducing activity of U937 cells, in the presence of 75 μM HU, it effectively induced this activity to 5.96 $A_{560}$, which is similar to the value (5.90 $A_{560}$) with a 100-fold greater concentration ($3 \times 10^{-8}$ M) of $1,25(OH)_2D_3$ (Figure 2B). Morphologically, monoblastic U937 cells were induced to differentiate into monocytic cells by $1,25(OH)_2D_3$ and became more mature, having abundant and grey cytoplasm and a chromatin-condensed nucleus with the addition of HU (data not shown). HU also effectively enhanced the expression of CD11b in U937 cells induced by low concentrations of $1,25(OH)_2D_3$ (Figure

**Figure 3** Effects of the combination of HU and $1,25(OH)_2D_3$ with regard to the NBT-reducing activity of human myeloid leukaemia HL-60 (**A**), ML-1 (**B**), THP-1 (**C**), P39/TSU (**D**), P31/FUJ (**E**) and NB4 cells (**F**). Cells ($5 \times 10^4$ cells $ml^{-1}$) were cultured with HU in combination with 0 (●), $3 \times 10^{-9}$ M (▲) or $3 \times 10^{-8}$ M (■) $1,25(OH)_2D_3$ for 4 days. Values represent the means ± SD of three separate experiments

2C). HU at 75 μM plus $1,25(OH)_2D_3$ at $3 \times 10^{-9}$ M increased this intensity to 8.00 units, which is greater than 6.41 units with $3 \times 10^{-6}$ M $1,25(OH)_2D_3$ alone. The enhancing effect of HU on CD14 expression induced by $1,25(OH)_2D_3$ in U937 cells was weak (data not shown). Thus, HU effectively enhanced several differentiation markers in U937 cells treated with $1,25(OH)_2D_3$.

Next, we examined the combination of HU and $1,25(OH)_2D_3$ on the differentiation of other myelomonocytic leukaemia cells. HU did not induce NBT-reducing activity in promyelocytic HL-60 and myeloblastic ML-1 cells, but enhanced the activity induced by $1,25(OH)_2D_3$ (Figure 3A and B). HU alone induced NBT-reducing activity in monoblastic THP-1 cells but not in other monoblastic P39/TSU or P31/FUJ cells. It also effectively enhanced the differentiation of these cells induced by $1,25(OH)_2D_3$ (Figure 3C–E). NB4 cells are promyelocytic leukaemia cells with a t(15;17) chromosomal translocation and have been reported to be resistant to $1,25(OH)_2D_3$ (Testa et al, 1994). HU also induced the NBT-reducing activity in NB4 cells in combination with $1,25(OH)_2D_3$ (Figure 3F). Thus, HU plus $1,25(OH)_2D_3$ effectively induces the differentiation of myelomonocytic leukaemia cells.

### Effects of HU in combination with vitamin D derivatives on growth inhibition and differentiation induction in U937 cells

24-*Epi*-$1,25(OH)_2D_2$ and $1,25(OH)_2D_7$ have been reported to exhibit less hypercalcaemic activity than $1,25(OH)_2D_3$ and to be able to induce the differentiation of HL-60 cells (Sato et al, 1991). They also induced the NBT-reducing activity of U937 cells, and HU effectively enhanced the activities induced by their suboptimal concentrations (Figure 4A). 24-*Epi*-$1,25(OH)_2D_2$ and 1,25-$(OH)_2D_7$ inhibited the proliferation of U937 cells concentration dependently, with $IC_{50}$ values of $2.25 \times 10^{-7}$ M and $2.28 \times 10^{-7}$ M respectively (data not shown). At a low concentration of $9 \times 10^{-9}$ M, they slightly inhibited the proliferation of U937 cells and augmented the growth inhibition in combination with 50 μM HU (Table 2).

We have previously reported that $1\alpha(OH)D_3$ induces the differentiation of monoblastic leukaemia cells as well as $1,25(OH)_2D_3$ and is less toxic than $1,25(OH)_2D_3$ (Honma et al, 1983; Okabe-Kado et al, 1992). We examined the effects of several $1\alpha(OH)D$ derivatives on

© Cancer Research Campaign 1998

**Table 2** Growth inhibition in human monoblastic leukaemia U937 cells by vitamin D derivatives in combination with HU

| Compounds | Growth (% of control) | | |
|---|---|---|---|
| | – HU | + HU | Ratio[a] |
| None | 100 | 54 ± 3 | 100 |
| 1,25(OH)$_2$D$_3$ (3 × 10$^{-9}$ M) | 74 ± 1 | 25 ± 3 | 46 |
| 24-*Epi*-1,25(OH)$_2$D$_2$ (9 × 10$^{-9}$ M) | 87 ± 2 | 33 ± 1 | 61 |
| 1,25(OH)$_2$D$_7$ (9 × 10$^{-9}$ M) | 89 ± 2 | 32 ± 1 | 59 |
| 1α(OH)D$_3$ (3 × 10$^{-8}$ M) | 72 ± 2 | 27 ± 1 | 50 |
| 1α(OH)D$_2$ (6 × 10$^{-8}$ M) | 91 ± 2 | 31 ± 1 | 57 |
| 1α(OH)D$_4$ (6 × 10$^{-8}$ M) | 89 ± 1 | 28 ± 3 | 52 |
| 1α(OH)D$_7$ (6 × 10$^{-8}$ M) | 100 ± 4 | 27 ± 2 | 50 |

Cells (5 × 10$^4$ cells ml$^{-1}$) were cultured with vitamin D derivatives in the absence or presence of 50 μM HU for 4 days. [a]Ratio (%) represents the growth of cells in combination with HU compared with that of cells treated with 50 μM HU alone.

growth inhibition and differentiation induction in U937 cells in combination with HU. 1α(OH)D$_3$ inhibited proliferation with an IC$_{50}$ value of 0.67 × 10$^{-7}$ M and induced myelomonocytic differentiation markers, such as the NBT-reducing and lysozyme activities of U937 cells (Figure 4B, data not shown). HU effectively enhanced the NBT-reducing activity in U937 cells induced by 1α(OH)D$_3$ (Figure 4B). For example, 1.2 × 10$^{-8}$ M 1α(OH)D$_3$ plus 50 μM HU induced this activity to 9.02 A$_{560}$, while this activity was 8.54 A$_{560}$ with 1.2 × 10$^{-6}$ M 1α(OH)D$_3$ alone, indicating that 1α(OH)D$_3$ was more than 100 times as active in the presence of HU. 1α(OH)D$_3$ at 3 × 10$^{-8}$ M slightly inhibited the proliferation of U937 cells, but augmented the inhibition in combination with HU (Table 2). 1α(OH)D$_2$, 1α(OH)D$_4$ and 1α(OH)D$_7$ also inhibited the proliferation with IC$_{50}$ values of 1.35 × 10$^{-7}$, 1.47 × 10$^{-7}$ and 3.25 × 10$^{-7}$ M, respectively (data not shown), and induced the differentiation of monoblastic U937 (Figure 4C), P39/TSU and P31/FUJ cells, but not of promyelocytic HL-60 cells (data not shown). Among these four 1α(OH)D derivatives, 1α(OH)D$_3$ was the most effective in inhibiting the proliferation of U937 cells ($P$<0.005, compared at IC$_{50}$). At the IC$_{50}$ values for growth inhibition, 1α(OH)D$_3$, 1α(OH)D$_2$, 1α(OH)D$_4$ and 1α(OH)D$_7$ induced NBT-reducing activity of U937 cells from 0.83 A$_{560}$ to 5.80, 5.50, 6.08 and 5.84 A$_{560}$, respectively, and lysozyme activity from 1.99 units to 5.03, 5.11, 6.34 and 6.03 units, respectively (data not shown), indicating that 1α(OH)D$_4$ was slightly more effective for inducing these activities than the others. The NBT-reducing activity induced by these 1α(OH)D derivatives in U937 cells was also effectively enhanced by HU (Figure 4C). HU enhanced the induction of NBT reduction by 1α(OH)D$_4$ slightly more effectively than that by 1α(OH)D$_2$ and 1α(OH)D$_7$. At a concentration of 6 × 10$^{-8}$ M, 1α(OH)D$_2$ and 1α(OH)D$_4$ inhibited the proliferation of U937 cells only slightly, while 1α(OH)D$_7$ had no such effect, but they augmented the growth-inhibitory activity with HU (Table 2). Thus, the combination of HU with 1α(OH)D derivatives is effective for inhibiting the proliferation and inducing the differentiation of U937 cells.

### DISCUSSION

Among the anti-cancer drugs we examined, HU showed the greatest synergistic effect with 1,25(OH)$_2$D$_3$ with regard to growth inhibition and differentiation induction in U937 cells. Ara-C and camptothecin showed modest synergism with regard to growth inhibition, and Ara-C enhanced the differentiation induced by 1,25(OH)$_2$D$_3$ second only to HU. In another study, treatment with

**Figure 4** Effects of vitamin D derivatives in combination with HU with regard to the induction of NBT-reducing activity in human monoblastic leukaemia U937 cells. (**A**) NBT-reducing activities of cells treated with 24-*epi*-1,25(OH)$_2$D$_2$ (○, ●) or 1,25(OH)$_2$D$_7$ (△, ▲) in the absence (○, △) or presence (●, ▲) of 50 μM HU. (**B**) NBT-reducing activities of cells treated with 1α(OH)D$_3$ in the absence (○) or presence (●) of 50 μM HU. (c) NBT-reducing activities of cells treated with 1α(OH)D$_2$ (○, ●), 1α(OH)D$_4$ (△, ▲) or 1α(OH)D$_7$ (□, ■) in the absence (○, △, □) or presence (●, ▲, ■) of 50 μM HU. Cells (5 × 10$^4$ cells ml$^{-1}$) were cultured with the test compounds for 4 days. Values represent the means ± SD of three separate experiments

© Cancer Research Campaign 1998

$1,25(OH)_2D_3$ increased the cytotoxicity of Ara-C and HU against HL-60 cells (Studzinski et al, 1986). HU inhibits nucleotide metabolism by inhibiting ribonucleotide reductase and Ara-C also inhibits nucleotide synthesis (Calabresi and Chabner, 1996). HU enhances the differentiation of HL-60 cells induced by all-*trans* retinoic acid (Yen et al, 1987). Ara-C induces the differentiation of some myeloid leukaemia cells and low doses of Ara-C have been used to treat acute myeloid leukaemia (Housset et al, 1982). Other inhibitors of nucleotide metabolism also induce the differentiation of myeloid leukaemia cells (Bodner et al, 1981; Ishiguro and Sartorelli, 1985). These findings indicate that some inhibitors of nucleotide metabolism may induce leukaemia cells to differentiate and to enhance differentiation induced by other compounds more effectively than other types of anti-cancer drugs.

HU is useful for treating chronic myelogenous leukaemia and, when administered orally at daily doses from 500 to 3000 mg, for controlling blood cell counts within desirable ranges (Athens, 1993). Its major adverse effect is bone marrow suppression, but the bone marrow recovers promptly if the drug is discontinued for a few days. Thus, HU can be used safely in elderly patients. Pharmacokinetic studies have shown that serum concentrations of HU after a single oral administration of 1000 mg reach 20–30 $\mu g\ ml^{-1}$ (263–394 $\mu$M) in 1–3 h, then gradually decrease and remain higher than 5 $\mu g\ ml^{-1}$ (66 $\mu$M) for at least 10 h (Davidson and Winter, 1963; Bolton et al, 1965). These findings indicate that the concentrations of HU needed to enhance the anti-leukaemic activity of vitamin D derivatives can be achieved clinically.

In this study, we observed the differentiation-inducing activities of $1\alpha(OH)D$ derivatives. After the administration of $1\alpha(OH)D_3$, it is converted to an active form, $1,25(OH)_2D_3$, by liver 25-hydroxylase (Holick et al, 1975). $1\alpha(OH)D_3$ was more potent than $1,25(OH)_2D_3$ in increasing the survival time of mice inoculated with mouse myeloid leukaemia M1 cells (Honma et al, 1983). The relatively stable concentrations of $1,25(OH)_2D_3$ after the administration of $1\alpha(OH)D_3$ compared with $1,25(OH)_2D_3$ may contribute to the advantage offered by $1\alpha(OH)D_3$. Interestingly, $1\alpha(OH)D_3$ induced the differentiation of monoblastic leukaemia cells in vitro and was converted to $1,25(OH)_2D_3$ in the cells (Okabe-Kado et al, 1992). As monocytes also have 24-hydroxylase activity (Kamimura et al, 1995) and $1\alpha$, 24-dihydroxyvitamin $D_3$ can induce the differentiation of HL-60 cells to a similar extent as $1,25(OH)_2D_3$ (Tanaka et al, 1982), $1\alpha(OH)D_3$ may act by being converted to $1,25(OH)_2D_3$ and $1\alpha$, 24-dihydroxyvitamin $D_3$ in monocytic cells. Thus, $1\alpha(OH)D_3$ affects leukaemia cells with monocytic characteristics both directly and indirectly. Acute monocytic leukaemia is more resistant to intensive chemotherapy than other types of acute myeloid leukaemia and its prognosis is poor (Fenaux et al, 1990). The use of $1\alpha(OH)D$ derivatives may offer certain advantages in the treatment of monocytic leukaemia, as (a) $1,25(OH)_2D_3$ is physiologically catabolized to an inactive form in monocytic cells and such cells can activate $1\alpha(OH)D$ and (b) drugs can be focused against leukaemia cells of monocytic lineage and their adverse effects against other organs can be diminished. Other $1\alpha(OH)D$ derivatives, including $1\alpha(OH)D_2$, $1\alpha(OH)D_4$ and $1\alpha(OH)D_7$, also induced the differentiation of myelomonocytic leukaemia cells. $1\alpha(OH)D_2$ is 5–15 times less toxic than $1\alpha(OH)D_3$ in rats (Sjöden et al, 1985). A clinical study in post-menopausal osteopenic patients showed that $1\alpha(OH)D_2$ at daily doses of less than 5.0 $\mu$g did not induce hypercalcaemia, whereas $1\alpha(OH)D_3$ at daily doses above 1.0 $\mu$g had toxic effects (Gallagher et al, 1994). An active form of $1\alpha(OH)D_7$, $1,25(OH)_2D_7$, has less hypercalcaemic activity (Sato et al, 1991). Therefore, the $1\alpha(OH)D$ derivatives may be useful for treating monocytic leukaemia. Pharmacokinetics for serum concentrations of $1\alpha(OH)D$ derivatives and their metabolites after administration should be further investigated. HU also effectively enhanced the differentiation induced by the $1\alpha(OH)D$ derivatives. The combination of $1\alpha(OH)D$ derivatives with HU may be a promising candidate for 'chemo-differentiation therapy' of acute monocytic leukaemia.

### ACKNOWLEDGEMENTS

This work was supported in part by Grants for Cancer Research from the Ministry of Education, Science and Culture of Japan. We thank Dr Yoji Tachibana (Fine Chemical Research Center, Nisshin Flour Milling, Saitama, Japan) for kindly providing vitamin D derivatives and for his helpful discussions.

### REFERENCES

Abe E, Miyaura C, Sakagami H, Takeda M, Konno K, Yamazaki T, Yoshiki S and Suda T (1981) Differentiation of mouse myeloid leukemia cells induced by $1\alpha$,25-dihydroxyvitamin $D_3$. *Proc Natl Acad Sci USA* **78**: 4990–4994

Abe J, Nakano T, Nishii Y, Matsumoto T, Ogata E and Ikeda K (1991) A novel vitamin $D_3$ analog, 22-oxa-1,25-dihydroxyvitamin $D_3$, inhibits the growth of human breast cancer *in vitro* and *in vivo* without causing hypercalcemia. *Endocrinology* **129**: 832–837

Athens JW (1993) Chronic myeloid leukaemia. In *Wintrobe's Clinical Hematology*, Lee GR, Bithell TC, Foerster J, Athens JW and Lukens JN. (eds), pp. 1969–1998. Lea & Febger: Philadelphia

Berenbaum MC (1989) What is synergy? *Pharmacol Rev* **41**: 93–141

Bolton BH, Woods LA, Kaung DT and Lawton RL (1965) A simple method of colorimetric analysis for hydroxyurea (NSV-32065). *Cancer Treat Rep* **46**: 1–5

Bodner AJ, Ting RC and Gallo RC (1981) Induction of differentiation of human promyelocytic leukemia cells (HL-60) by nucleosides and methotrexate. *J Natl Cancer Inst* **67**: 1025–1030

Calabresi P, Chabner BA (1996) Chemotherapy of neoplastic diseases. In *Goodman & Gilman's The Pharmacological Basis of Therapeutics*, 9th edn, Hardman JG, Limbird LE, Molinoff PB, Ruddon RW and Gilman AG. (eds), pp. 1225–1287. McGraw-Hill: New York

Davidson JD and Winter TS (1963) A method of analyzing for hydroxyurea in biological fluids. *Cancer Chemother Rep* **27**: 97–110

Degos L, Dombret H, Chomienne C, Daniel M-T, Micléa J-M, Chastang C, Castaigne S and Fenaux P (1995) All-*trans*-retinoic acid as a differentiating agent in the treatment of acute promyelocytic leukemia. *Blood* **85**: 2643–2653

Fenaux P, Vanhaesbroucke C, Estienne MH, Preud'Homme C, Pagniez D, Facon T, Millot F and Bauters F (1990) Acute monocytic leukaemia in adults: treatment and prognosis in 99 cases. *Br J Haematol* **75**: 41–48

Gallagher JC, Bishop CW, Knutson JC, Mazess RB and DeLuca HF (1994) Effects of increasing doses of $1\alpha$-hydroxyvitamin $D_2$ on calcium homeostasis in postmenopausal osteopenic women. *J Bone Miner Res* **9**: 607–614

Goldman JM (1994) A special report: bone marrow transplants using volunteer donors – recommendations and requirements for a standardized practice throughout the world – 1994 update. *Blood* **84**: 2833–2839

Holick MF, Holick SA, Tavela T, Gallagher B, Schonoes HK and DeLuca HF (1975) Synthesis of [6-$^3$H]-$1\alpha$-hydroxyvitamin $D_3$ and its metabolism in vivo to [$^3$H]-$1\alpha$,25-dihydroxyvitamin $D_3$. *Science* **190**: 576–578

Honma Y, Hozumi M, Abe E, Konno K, Fukushima M, Hata S, Nichii Y, DeLuca HF and Suda T (1983) $1\alpha$,25-Dihydroxyvitamin $D_3$ and $1\alpha$-hydroxyvitamin $D_3$ prolong survival time of mice inoculated with myeloid leukemia cells. *Proc Natl Acad Sci USA* **80**: 201–204

Housset M, Daniel MT and Degos L (1982) Small doses of Ara-C in the treatment of acute myeloid leukaemia: differentiation of myeloid leukaemia cells? *Br J Haematol* **51**: 125–129

Ishiguro K and Sartorelli AC (1985) Enhancement of the differentiation-inducing properties of 6-thioguanine by hypoxanthine and its nucleosides in HL-60 promyelocytic leukemia cells. *Cancer Res* **45**: 91–95

Kamimura S, Gallieni M, Zhong M, Beron W, Slatopolsky E and Dusso A (1995) Microtubules mediate cellular 25-hydroxyvitamin $D_3$ trafficking and the

© Cancer Research Campaign 1998

genomic response to 1,25-dihydroxyvitamin $D_3$ in normal human monocytes. *J Biol Chem* **270**: 22160–22166

Koeffler HP, Hirji K, Itri L, The Southern California Leukemia Group (1985) 1,25-Dihydroxyvitamin $D_3$: in vivo and in vitro effects on human preleukemic and leukemic cells. *Cancer Treat Rep* **69**: 1399–1407

Lanotte M, Martin-Thouvenin V, Najman S, Balerini P, Valensi F and Berger R (1991) NB4, a maturation inducible cell line with t(15;17) marker isolated from a human acute promyelocytic leukemia (M3). *Blood* **77**: 1080–1086

Makishima M and Honma Y (1996) Ethacrynic acid and 1α,25-dihydroxyvitamin $D_3$ cooperatively inhibit proliferation and induce differentiation of human myeloid leukemia cells. *Leuk Res* **20**: 781–789

Makishima M, Kanatani Y, Yamamoto-Yamaguchi Y and Honma Y (1996) Enhancement of activity of 1α,25-dihydroxyvitamin $D_3$ for growth inhibition and differentiation induction of human myelomonocytic leukemia cells by tretinoin tocoferil, an α-tocopherol ester of all-*trans* retinoic acid. *Blood* **87**: 3384–3394

Miyaura C, Abe E, Kuribayashi T, Tanaka H, Konno K, Nishii Y and Suda T (1981) 1α,25-Dihydroxyvitamin $D_3$ induces differentiation of human myeloid leukemia cells. *Biochem Biophys Res Commun* **102**: 937–943

Okabe-Kado J, Honma Y, Kasukabe T and Hozumi M (1992) Synthesis of active metabolite(s) from 1α-hydroxyvitamin $D_3$ by human monocytic leukemia cells. *FEBS Lett* **309**: 399–401

Overton WR (1988) Modified histogram subtraction technique for analysis of flow cytometry data. *Cytometry* **9**: 619–626

Pakkala S, De Vos S, Elstner E, Rude RK, Uskokovic M, Binderup L and Koeffler HP (1995) Vitamin $D_3$ analogs: effect on leukemic clonal growth and differentiation, and on serum calcium levels. *Leuk Res* **19**: 65–72

Sato F, Okamoto Y, Ouchi Y, Kaneki M, Nakamura T, Ikekawa N and Orimo H (1991) Biological activity of 1α,25-dihydroxyvitamin D derivatives – 24-*epi*-1α,25-dihydroxyvitamin D-2 and 1α,25-dihydroxyvitamin D-7. *Biochim Biophys Acta* **1091**: 188–192

Schiffer CA (1996) Hematopoietic growth factors as adjuncts to the treatment of acute myeloid leukemia. *Blood* **88**: 3675–3685

Sjöden G, Smith C, Lindgren U and DeLuca HF (1985) 1α-hydroxyvitamin $D_2$ is less toxic than 1α-hydroxyvitamin $D_3$ in the rat. *Proc Soc Exp Biol Med* **178**: 432–436

Steel GG and Peckham MJ (1979) Exploitable mechanisms in combined radiotherapy–chemotherapy: the concept of additivity. *Int J Radiat Oncol Biol Phys* **5**: 85–91

Studzinski GP, Bhandal AK and Brelvi ZS (1986) Potentiation by 1-α,25-dihydroxyvitamin $D_3$ of cytotoxicity to HL-60 cells produced by cytarabine and hydroxyurea. *J Natl Cancer Inst* **76**: 641–648

Tachibana Y and Tsuji M (1992) Synthetic studies on active forms of vitamin D and their analogs. In *Studies in Natural Products Chemistry*, Vol. 11, Rahman AU. (ed.), pp. 379–408. Elsevier: Amsterdam

Tanaka H, Abe E, Miyaura C, Kuribayashi T, Konno K, Nishii Y and Suda T (1982) 1α,25-Dihydroxycholecalciferol and a human myeloid leukaemia cell line (HL-60). The presence of a cytosol receptor and induction of differentiation. *Biochem J* **204**: 713–719

Testa U, Grignani F, Barberi T, Fagioli M, Masciulli R, Ferrucci PF, Seripa D, Camagna A, Alcalay M, Pelicci PG and Peschle C (1994) PML/RARα+ U937 mutant and NB4 cell lines: retinoic acid restores the monocytic differentiation response to vitamin $D_3$. *Cancer Res* **54**: 4508–4515

Venditti A, Stasi R, Del Poeta G, Buccisano F, Aronica G, Bruno A, Pisani F, Caravita T, Masi M, Tribalto M, Simone MD, Avvisati G, Amadori S and Papa G (1995) All-*trans* retinoic acid and low-dose cytosine arabinoside for the treatment of 'poor prognosis' acute myeloid leukemia. *Leukemia* **9**: 1121–1125

Yen A, Freeman L and Fishbaugh J (1987) Hydroxyurea induces precommitment during retinoic induced HL-60 terminal myeloid differentiation: possible involvement of gene amplification. *Leuk Res* **11**: 63–71

© Cancer Research Campaign 1998


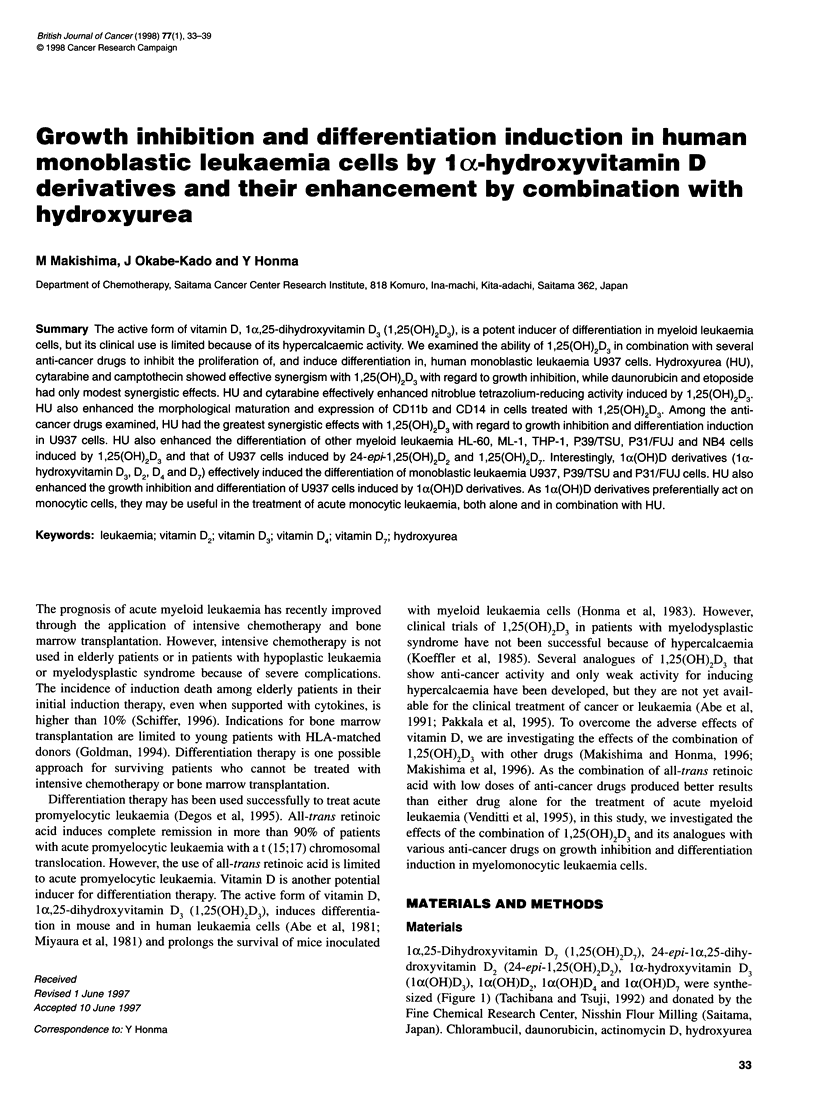

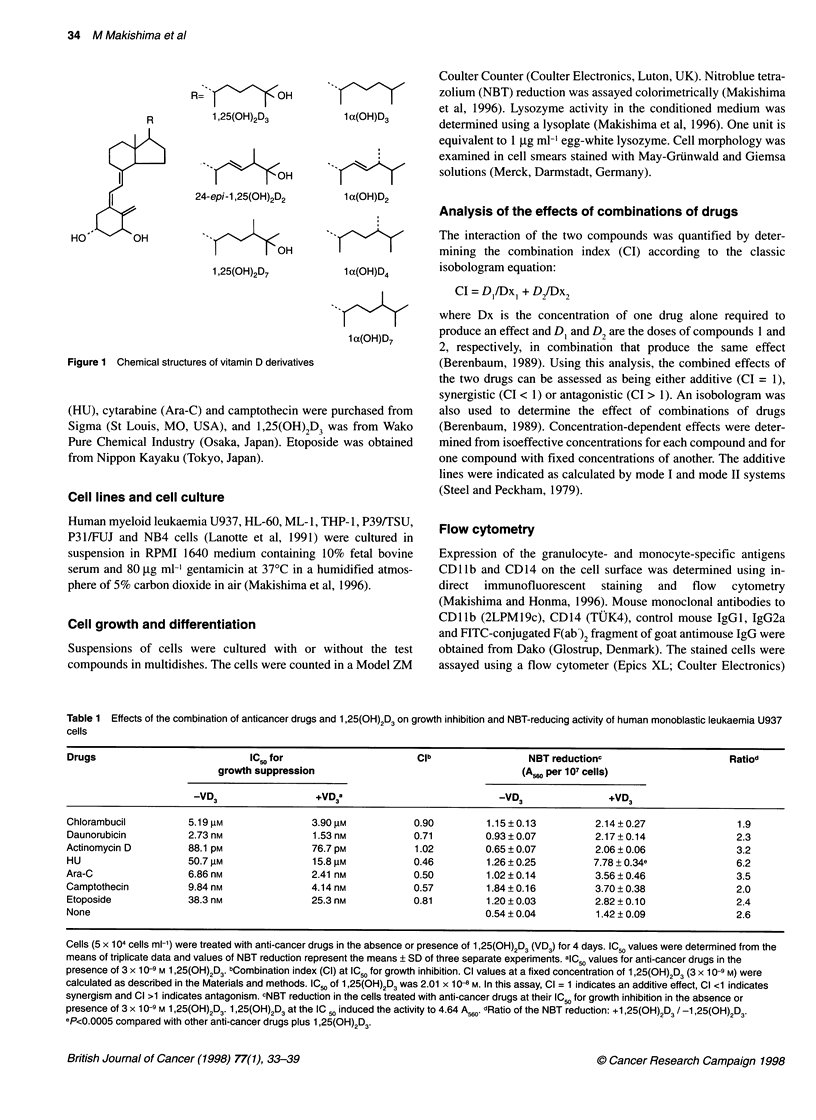

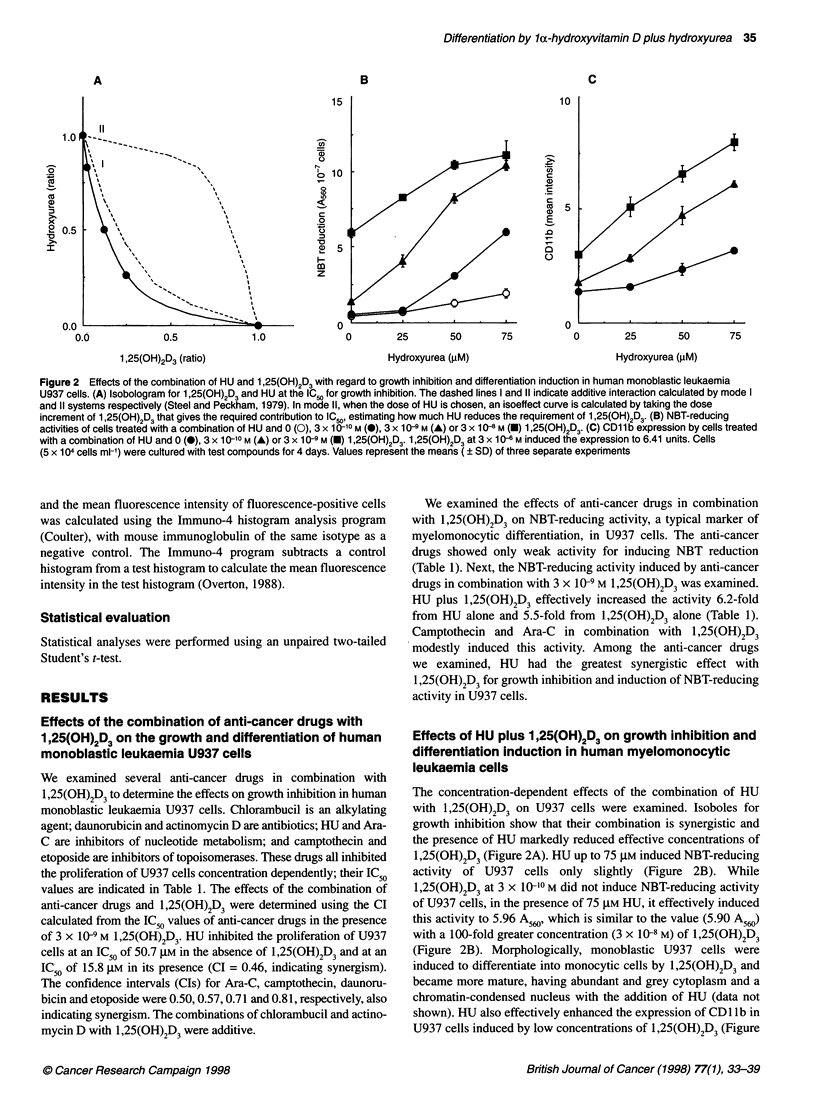

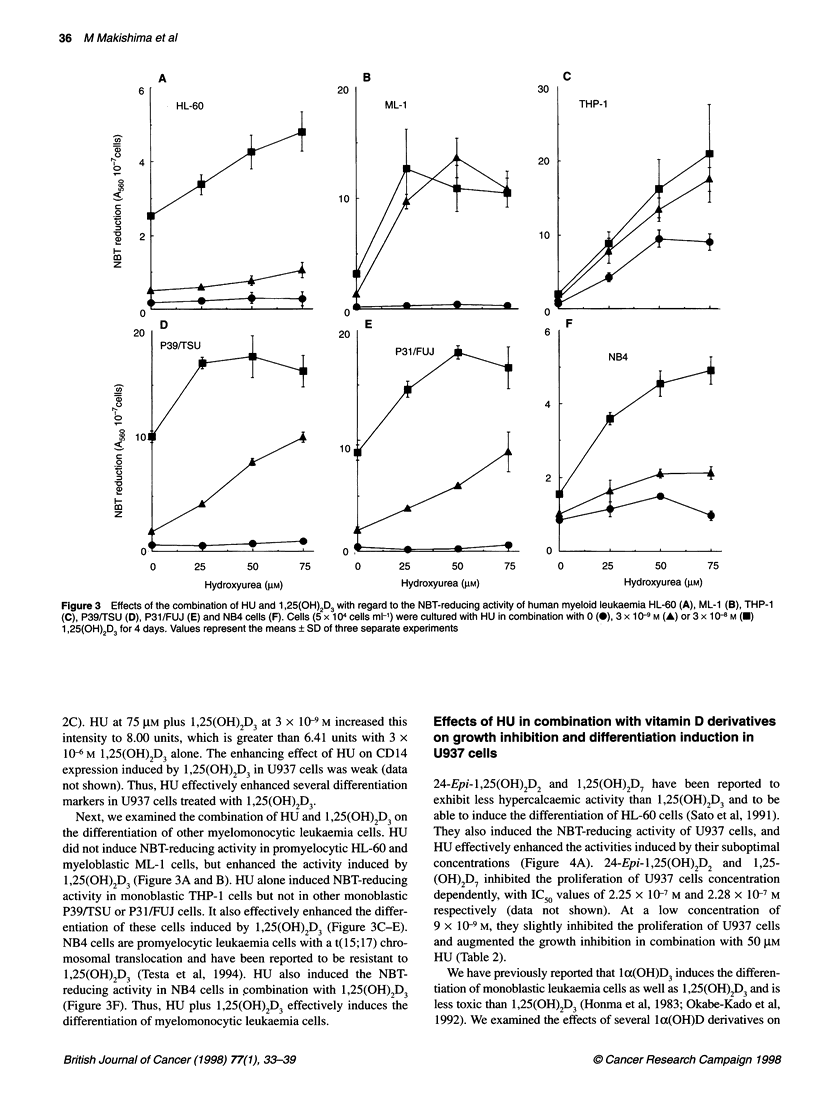

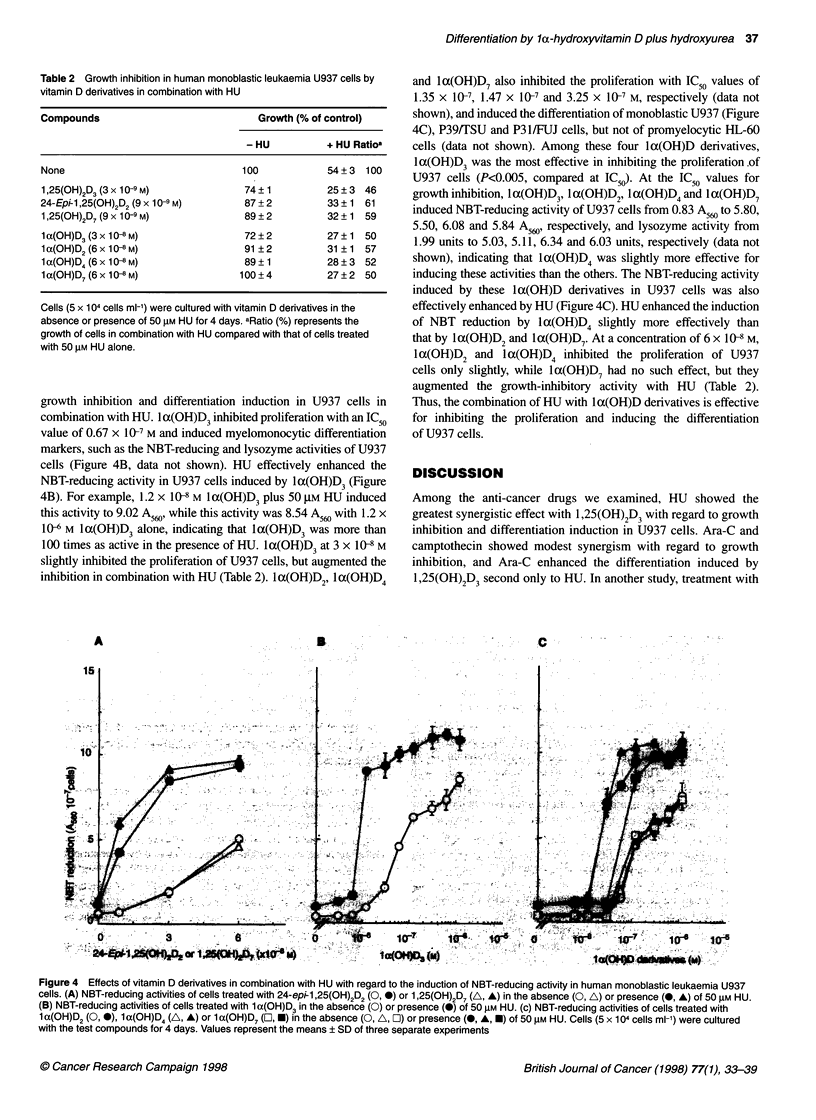

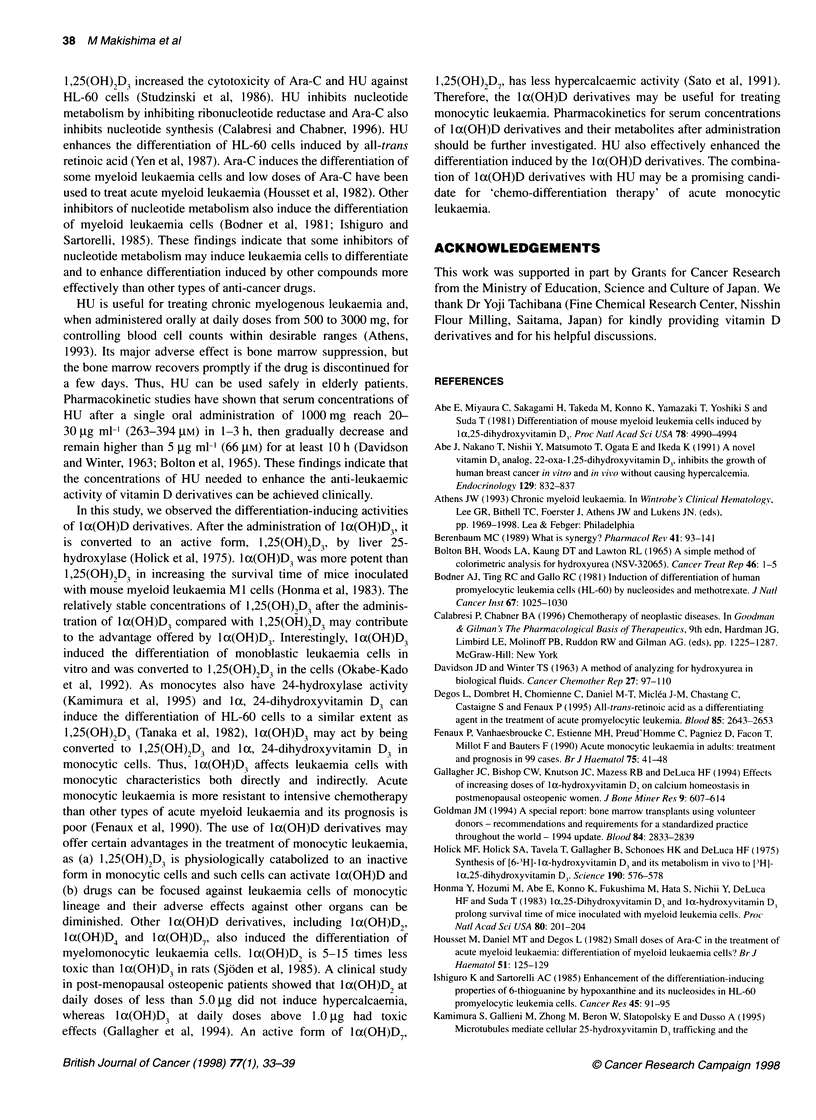

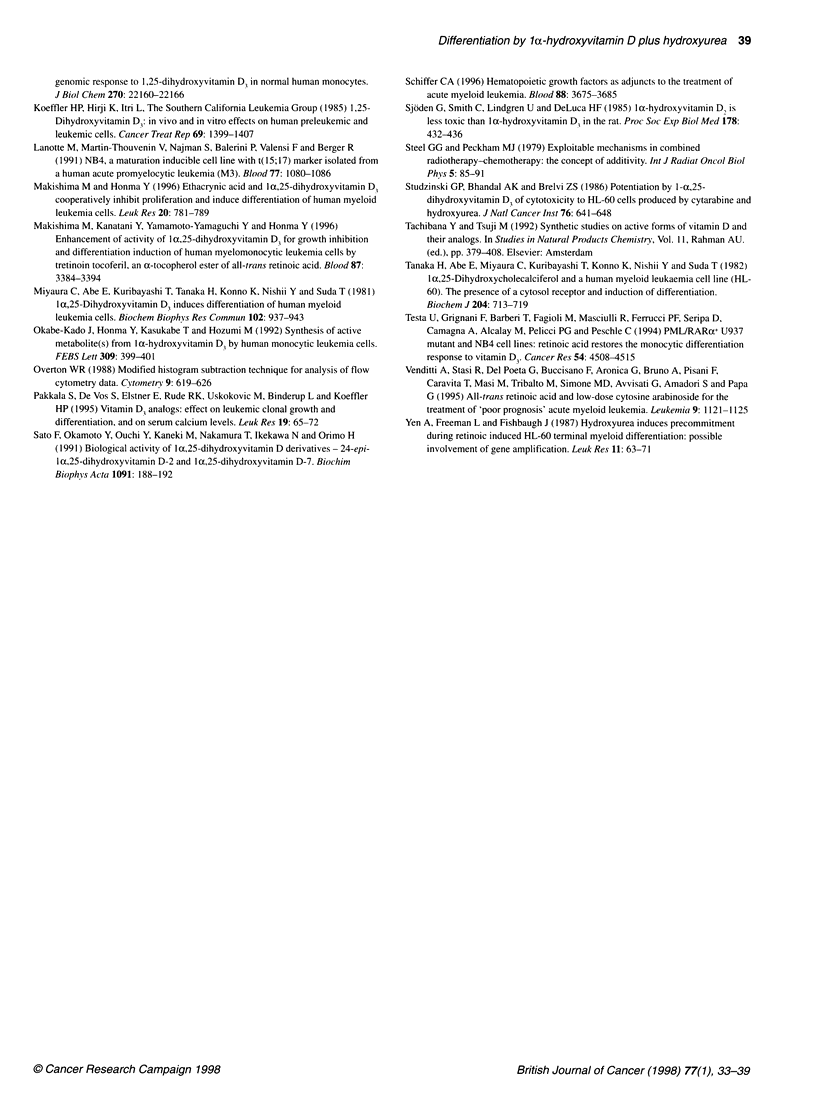


## References

[OCR_00681] Abe E., Miyaura C., Sakagami H., Takeda M., Konno K., Yamazaki T., Yoshiki S., Suda T. (1981). Differentiation of mouse myeloid leukemia cells induced by 1 alpha,25-dihydroxyvitamin D3.. Proc Natl Acad Sci U S A.

[OCR_00684] Abe J., Nakano T., Nishii Y., Matsumoto T., Ogata E., Ikeda K. (1991). A novel vitamin D3 analog, 22-oxa-1,25-dihydroxyvitamin D3, inhibits the growth of human breast cancer in vitro and in vivo without causing hypercalcemia.. Endocrinology.

[OCR_00697] BOLTON B. H., WOODS L. A., KAUNG D. T., LAWTON R. L. (1965). A SIMPLE METHOD OF COLORIMETRIC ANALYSIS FOR HYDROXYUREA (NSC-32065).. Cancer Chemother Rep.

[OCR_00695] Berenbaum M. C. (1989). What is synergy?. Pharmacol Rev.

[OCR_00700] Bodner A. J., Ting R. C., Gallo R. C. (1981). Induction of differentiation of human promyelocytic leukemia cells (HL-60) by nucleosides and methotrexate.. J Natl Cancer Inst.

[OCR_00715] Degos L., Dombret H., Chomienne C., Daniel M. T., Micléa J. M., Chastang C., Castaigne S., Fenaux P. (1995). All-trans-retinoic acid as a differentiating agent in the treatment of acute promyelocytic leukemia.. Blood.

[OCR_00722] Fenaux P., Vanhaesbroucke C., Estienne M. H., Preud'homme C., Pagniez D., Facon T., Millot F., Bauters F. (1990). Acute monocytic leukaemia in adults: treatment and prognosis in 99 cases.. Br J Haematol.

[OCR_00725] Gallagher J. C., Bishop C. W., Knutson J. C., Mazess R. B., DeLuca H. F. (1994). Effects of increasing doses of 1 alpha-hydroxyvitamin D2 on calcium homeostasis in postmenopausal osteopenic women.. J Bone Miner Res.

[OCR_00730] Goldman J. M. (1994). A special report: bone marrow transplants using volunteer donors--recommendations and requirements for a standardized practice throughout the world--1994 update. The WMDA Executive Committee.. Blood.

[OCR_00735] Holick M. F., Holick S. A., Tavela T., Gallagher B., Schnoes H. K., DeLuca H. F. (1975). Synthesis of (6-3H)-1alpha-hydroxyvitamin D3 and its metabolism in vivo to (3H)-1alpha,25-dihydroxyvitamin D3.. Science.

[OCR_00740] Honma Y., Hozumi M., Abe E., Konno K., Fukushima M., Hata S., Nishii Y., DeLuca H. F., Suda T. (1983). 1 alpha,25-Dihydroxyvitamin D3 and 1 alpha-hydroxyvitamin D3 prolong survival time of mice inoculated with myeloid leukemia cells.. Proc Natl Acad Sci U S A.

[OCR_00746] Housset M., Daniel M. T., Degos L. (1982). Small doses of ARA-C in the treatment of acute myeloid leukaemia: differentiation of myeloid leukaemia cells?. Br J Haematol.

[OCR_00751] Ishiguro K., Sartorelli A. C. (1985). Enhancement of the differentiation-inducing properties of 6-thioguanine by hypoxanthine and its nucleosides in HL-60 promyelocytic leukemia cells.. Cancer Res.

[OCR_00756] Kamimura S., Gallieni M., Zhong M., Beron W., Slatopolsky E., Dusso A. (1995). Microtubules mediate cellular 25-hydroxyvitamin D3 trafficking and the genomic response to 1,25-dihydroxyvitamin D3 in normal human monocytes.. J Biol Chem.

[OCR_00767] Koeffler H. P., Hirji K., Itri L. (1985). 1,25-Dihydroxyvitamin D3: in vivo and in vitro effects on human preleukemic and leukemic cells.. Cancer Treat Rep.

[OCR_00772] Lanotte M., Martin-Thouvenin V., Najman S., Balerini P., Valensi F., Berger R. (1991). NB4, a maturation inducible cell line with t(15;17) marker isolated from a human acute promyelocytic leukemia (M3).. Blood.

[OCR_00777] Makishima M., Honma Y. (1996). Ethacrynic acid and 1 alpha,25-dihydroxyvitamin D3 cooperatively inhibit proliferation and induce differentiation of human myeloid leukemia cells.. Leuk Res.

[OCR_00782] Makishima M., Kanatani Y., Yamamoto-Yamaguchi Y., Honma Y. (1996). Enhancement of activity of 1alpha, 25-dihydroxyvitamin D3 for growth inhibition and differentiation induction of human myelomonocytic leukemia cells by tretinoin tocoferil, an alpha-tocopherol ester of all-trans retinoic acid.. Blood.

[OCR_00795] Okabe-Kado J., Honma Y., Kasukabe T., Hozumi M. (1992). Synthesis of active metabolite(s) from 1 alpha-hydroxyvitamin D3 by human monocytic leukemia cells.. FEBS Lett.

[OCR_00800] Overton W. R. (1988). Modified histogram subtraction technique for analysis of flow cytometry data.. Cytometry.

[OCR_00804] Pakkala S., de Vos S., Elstner E., Rude R. K., Uskokovic M., Binderup L., Koeffler H. P. (1995). Vitamin D3 analogs: effect on leukemic clonal growth and differentiation, and on serum calcium levels.. Leuk Res.

[OCR_00809] Sato F., Okamoto Y., Ouchi Y., Kaneki M., Nakamura T., Ikekawa N., Orimo H. (1991). Biological activity of 1 alpha, 25-dihydroxyvitamin D derivatives--24-epi-1 alpha, 25-dihydroxyvitamin D-2 and 1 alpha,25-dihydroxyvitamin D-7.. Biochim Biophys Acta.

[OCR_00815] Schiffer C. A. (1996). Hematopoietic growth factors as adjuncts to the treatment of acute myeloid leukemia.. Blood.

[OCR_00819] Sjöden G., Smith C., Lindgren U., DeLuca H. F. (1985). 1 alpha-Hydroxyvitamin D2 is less toxic than 1 alpha-hydroxyvitamin D3 in the rat.. Proc Soc Exp Biol Med.

[OCR_00824] Steel G. G., Peckham M. J. (1979). Exploitable mechanisms in combined radiotherapy-chemotherapy: the concept of additivity.. Int J Radiat Oncol Biol Phys.

[OCR_00829] Studzinski G. P., Bhandal A. K., Brelvi Z. S. (1986). Potentiation by 1-alpha,25-dihydroxyvitamin D3 of cytotoxicity to HL-60 cells produced by cytarabine and hydroxyurea.. J Natl Cancer Inst.

[OCR_00839] Tanaka H., Abe E., Miyaura C., Kuribayashi T., Konno K., Nishii Y., Suda T. (1982). 1 alpha,25-Dihydroxycholecalciferol and a human myeloid leukaemia cell line (HL-60).. Biochem J.

[OCR_00845] Testa U., Grignani F., Barberi T., Fagioli M., Masciulli R., Ferrucci P. F., Seripa D., Camagna A., Alcalay M., Pelicci P. G. (1994). PML/RAR alpha+ U937 mutant and NB4 cell lines: retinoic acid restores the monocytic differentiation response to vitamin D3.. Cancer Res.

[OCR_00851] Venditti A., Stasi R., Del Poeta G., Buccisano F., Aronica G., Bruno A., Pisani F., Caravita T., Masi M., Tribalto M. (1995). All-trans retinoic acid and low-dose cytosine arabinoside for the treatment of 'poor prognosis' acute myeloid leukemia.. Leukemia.

[OCR_00857] Yen A., Freeman L., Fishbaugh J. (1987). Hydroxyurea indices precommitment during retinoic induced HL-60 terminal myeloid differentiation: possible involvement of gene amplification.. Leuk Res.

